# Supplementary material for: A proteomic analysis of the PHF-forming tau fragment (tau297–391) following uptake into differentiated human neuronal SHSY5Y cells
Source: Biosci Rep. 2026 Sep 3;46(9):BSR20260349. doi: 10.1042/BSR20260349 (PMC13542910; doi:10.1042/BSR20260349)
Supplement: Supplementary file 1 [file BSR-2026-0349-T_supp.pdf]

Supplementary information

A proteomic analysis of the PHF-forming tau fragment (tau297-391) following uptake into differentiated human neuronal SHSY5Y cells

Alice Copsey[1], Sebastian S. Oakley[1], Mahmoud B. Maina[1,2], Robert Parker[3], Karen E. Marshall[1], Charles R. Harrington[4,5], John M.D. Storey[5,6], Claude M. Wischik[4,5], Louise C. Serpell [1]

[1] Sussex Neuroscience, School of Life Sciences, University of Sussex, Falmer, East Sussex, UK

[2] Biomedical Science Research and Training Centre, Yobe State University, Damaturu, Yobe State, Nigeria

[3] Centre for Immuno-Oncology, Nuffield Department of Medicine, University of Oxford, Oxford, OX3 7DQ, UK

[4] Institute of Medicine, Medical Sciences and Nutrition, University of Aberdeen, Aberdeen, UK

[5] TauRx Therapeutics Ltd., Aberdeen, UK

[6] Department of Chemistry, University of Aberdeen, Aberdeen, UK

***To whom correspondence should be addressed Louise C Serpell (L.C.Serpell@sussex.ac.uk)**

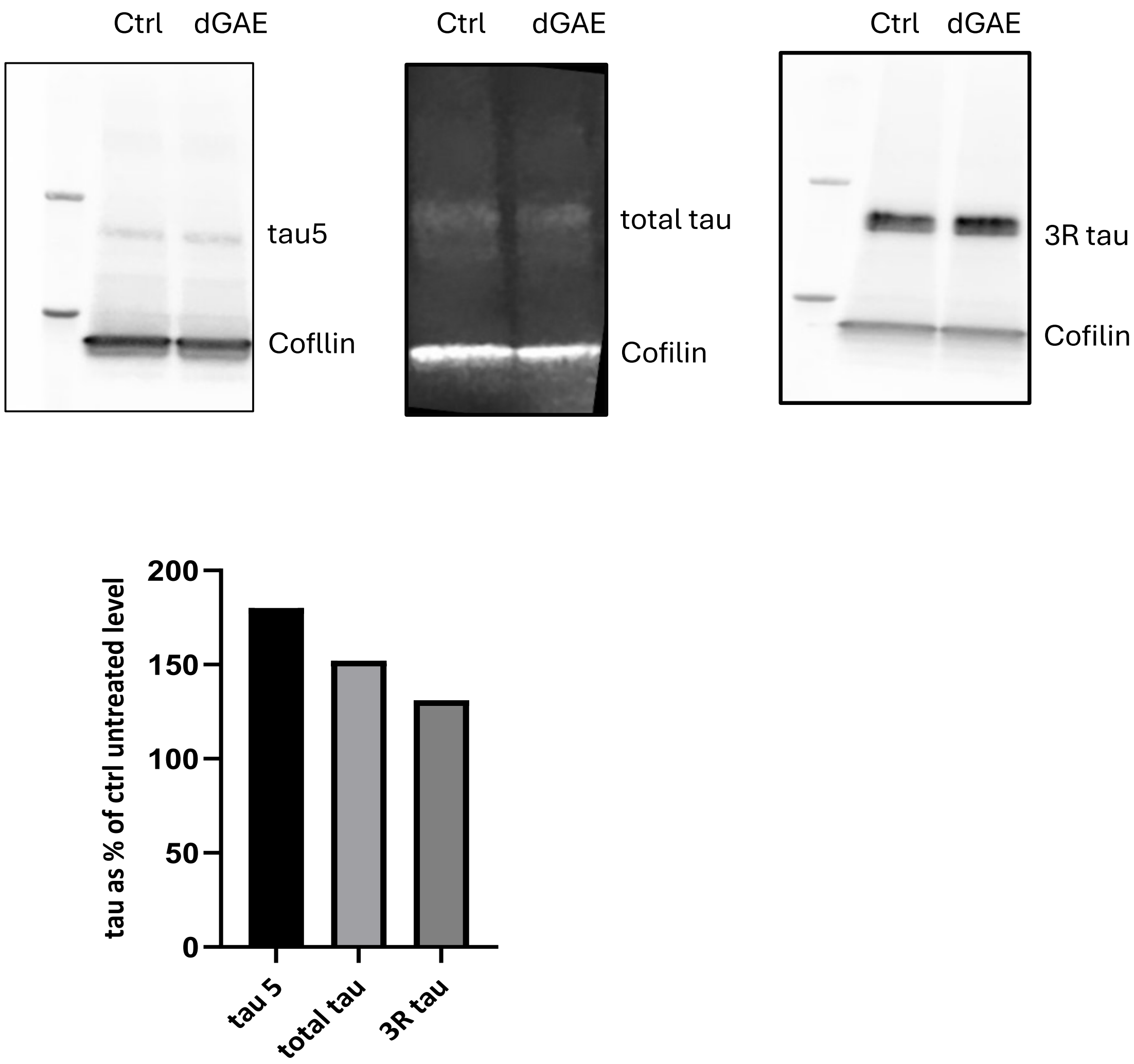

Supplementary Figure 1. Tau expression levels. *Protein lysates were prepared from dSH cells 24h post incubation with 10µM dGAE, alongside untreated control cells. Protein was lysed using RIPA buffer, separated by SDS-PAGE and visualised by immunoblotting with antibodies to tau (MAPT) and cofilin. Levels of tau expression were quantified relative to cofilin loading control and plotted using GraphPad Prism.*

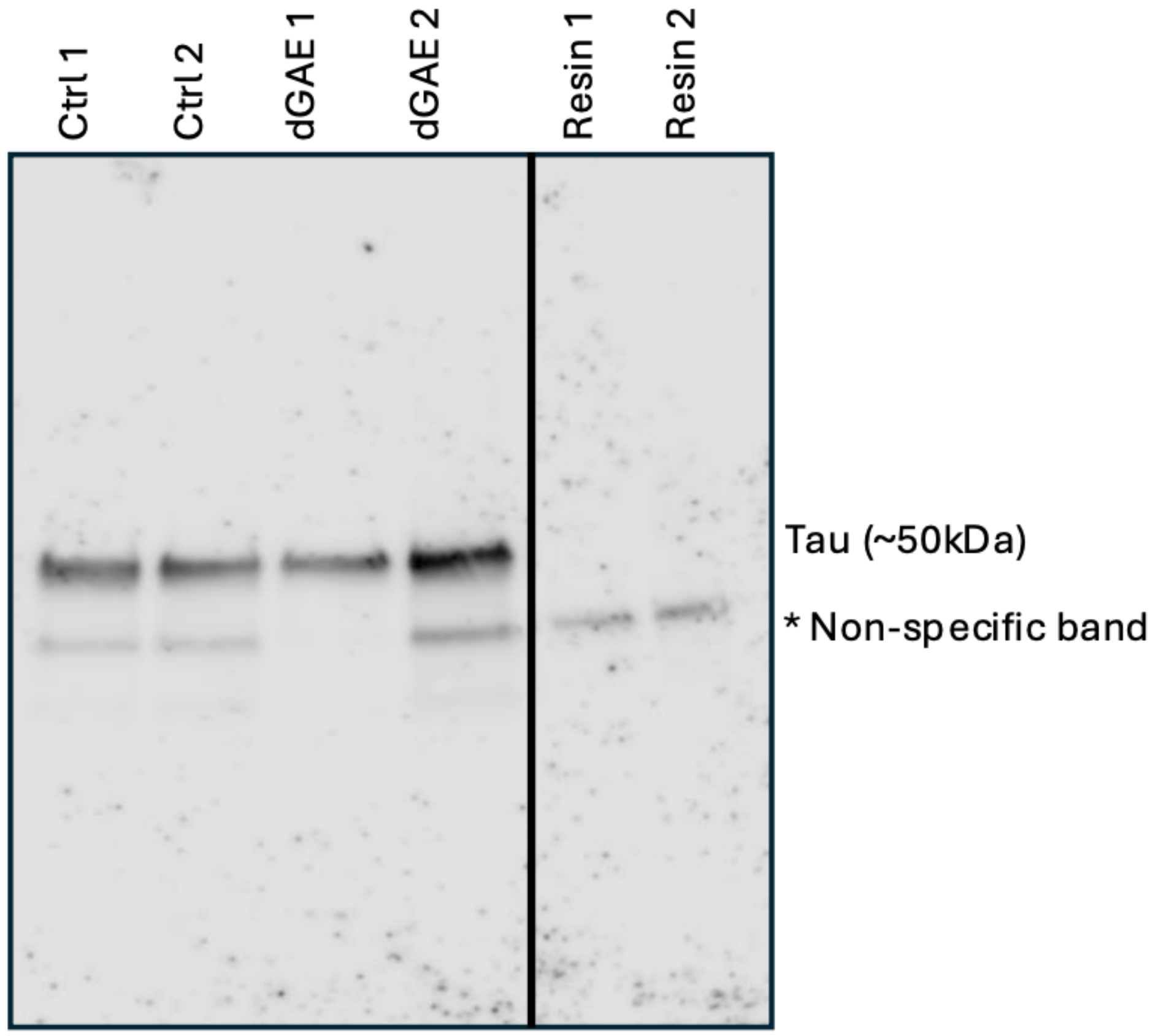

Supplementary Figure 2. Tau co-immunoprecipitation. *Tau and co-precipitating proteins were purified from whole cell lysates using antibodies to total tau (Rabbit, SAB4501831, Sigma) as described in the materials and method section. Eluted protein from control untreated, 10µM dGAE treated (24h) and resin only samples was separated by SDS-PAGE and visualised by immunoblotting with antibodies to tau (Mouse MAB3420, Chemicon). The image has been cropped and arranged to remove lanes which were not relevant to this study, a vertical line shows bringing together of two images of the same membrane.*

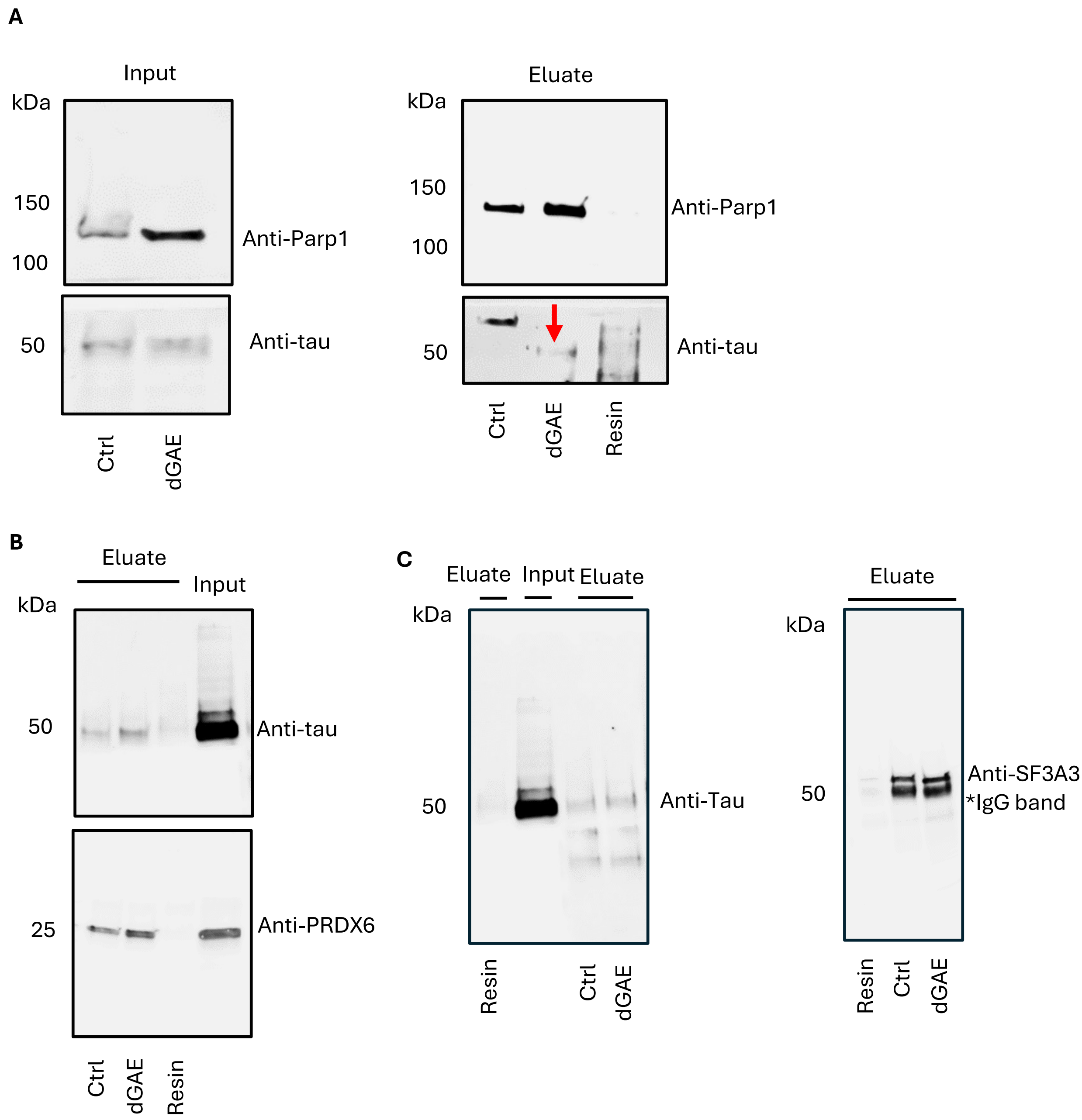

Supplementary Figure 3. Tau co-purifies with parp1, SF3A3 and PRDX6. *Protein lysates were prepared for immunoprecipitation from dSH cells 24h post incubation with 10µM dGAE, alongside untreated control cells. Cells were lysed in IP buffer and incubated with either parp1 antibody (46D11 cell Signalling), PRDX6 antibody (Proteintech 13585-1-AP) or SF3A3 antibody (Proteintech 12070-1-AP) as described in the materials and methods section. Precipitates from control untreated, dGAE treated and resin controls were eluted into 7M Urea, 2M Thio-urea, separated by SDS-PAGE and visualised by western blotting using antibodies to parp1 (46D11 cell Signalling) PRDX6 (Proteintech 13585-1-AP), SF3A3 (Proteintech 12070-1-AP) and tau (Mouse, MAB3420, Chemicon). Input samples are shown for antibody controls. A. Parp1 immunoprecipitation. B. PRDX6 immunoprecipitation. C. SF3A3 immunoprecipitates. Red arrow indicates tau band in dGAE sample for Parp1 immunoprecipitation.*

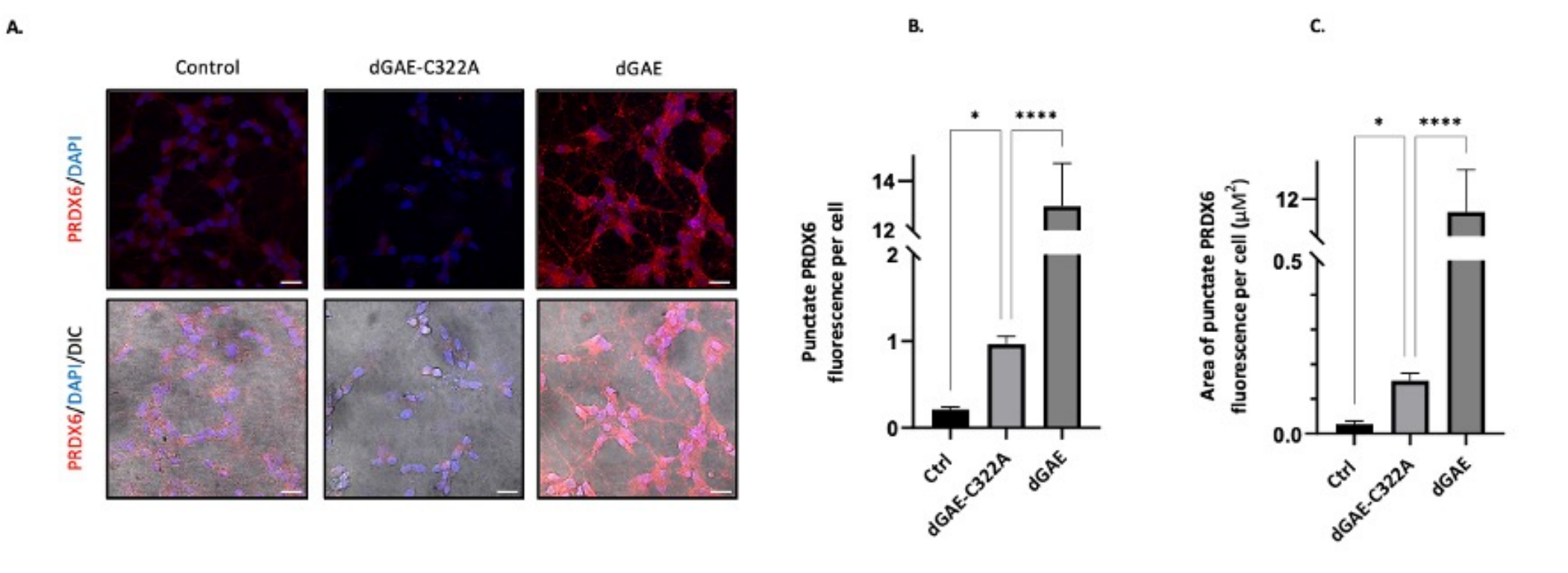

Supplementary figure 4. PRDX6 response is not observed with the treatment of dGAE-C322A. *Confocal images of dSH cells immunolabelled for PRDX6 (red) without treatment and the treatment of 10μM dGAE-C322A or 10μM dGAE for 24h. The Z-stack has been compiled using the Z-project function in FIJI with maximum intensity projection type. (b) Quantification and comparison of the particle count of punctate PRDX6 fluorescence per cells in control, dGAE-C322A and dGAE treated cells. Kruskal-Wallis ANOVA shows there is a significant difference between the groups (p < 0.0001). Dunn's multiple comparison test shows a significant difference between dGAE-C322A treated (0.9637 ± 0.09505) when compared to the control (0.2112 ± 0.02975, p = 0.0317) and dGAE treated (12.98 ± 1.737, p < 0.0001). (c) Quantification and comparison of the area of punctate PRDX6 fluorescence per cell for control, dGAE-C322A and dGAE treated cells. Kruskal-Wallis ANOVA shows there is a significant difference between the groups (p < 0.0001). Dunn's multiple comparison test shows a significant difference in dGAE-C322A treated (0.1523μM2 ± 0.02162μM2) when compared to the control (0.02699μM2 ± 0.009585μM2, p = 0.0467) and dGAE sample (11.65μM2 ± 1.143μM2, p < 0.0001). Confocal analysis comes from 4 independent tests for control (1162 cells) and dGAE-C322A (982 cells), 6 independent tests for dGAE (1964 cells).*

A

B

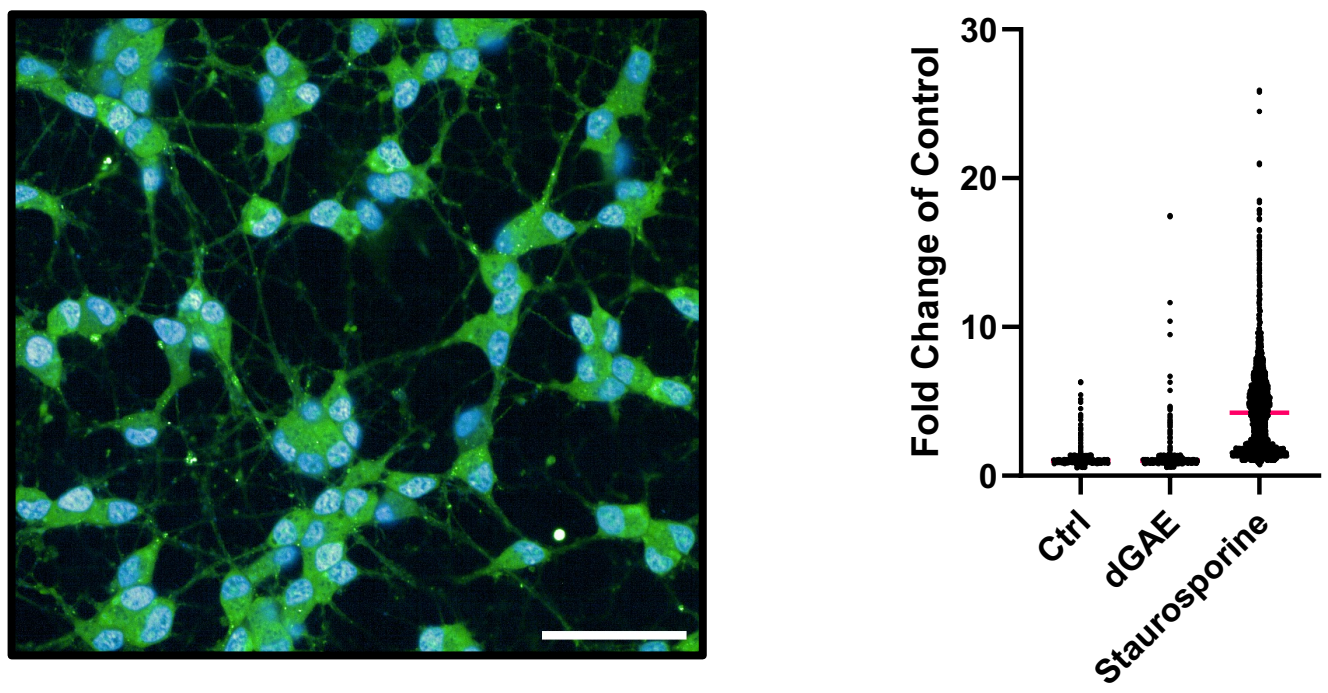

Supplementary Figure 5. *Positive control to show the effect of staurosporine. A) Image of dSH cells treated with 7μM staurosporine for 1h prior to* CellROX™ Green Reagent treatment. *B) Quantification of 24h treatment with positive control. Untreated control (1.003 ± 0.0009), dGAE (1.021 ± 0.0012) and staurosporine (4.244 ± 0.025) Analysis is from 3 independent experiments, control = 36256 cells, dGAE = 41884 cells and staurosporine = 13462.*

Majority protein IDs	Protein names	Gene names	Fold Change_dGAE high	Student's T-test p-value Control_dGAE	Abbreviated Function from Uniprot
P30041	Peroxiredoxin-6	PRDX6	353.3432943	0.024415392	Cell Protecton against oxidative stress
Q9ULV4	Coronin-1C	CORO1C	13.99642542	0.018034165	Cell Migration
P50897	Palmitoyl-protein thioesterase 1	PPT1	10.92179092	0.008401199	Lysosomal dagradation
Q12874	Splicing factor 3A subunit 3	SF3A3	9.603687689	0.049853139	pre-mRNA splicing
Q9UKV3	Apoptotic chromatin condensation inducer in the nucleus	ACIN1	7.862684785	0.015562521	Splicing process
Q13813	Spectrin alpha chain, non-erythrocytic 1	SPTAN1	7.500369081	0.026762826	Interacts with Calmodulin
Q09028	Histone-binding protein RBBP4	RBBP4	7.457239342	0.021270854	Chromatin metabolism
Q04726	Transducin-like enhancer protein 3	TLE3	7.176033607	0.031765715	Transcriptional corepressor
P31942	Heterogeneous nuclear ribonucleoprotein H3	HNRNPH3	6.887894195	0.004972538	Splicing process
Q02809	Procollagen-lysine,2-oxoglutarate 5-dioxygenase 1	PLOD1	6.105175876	0.047795036	Assembly and cross-linking of Collagen fibrils
Q7RTV0	PHD finger-like domain-containing protein 5A	PHF5A	5.804122238	0.030886588	component of the splicing factor SF3B complex
P22087	rRNA 2-O-methyltransferase fibrillarin	FBL	5.301076665	0.016193117	RNA and protein methyltransferase
Q9Y3Y2	Chromatin target of PRMT1 protein	CHTOP	4.956883053	0.004195265	Includes associates with spliced mRNA as part of TREX comlex
Q9Y5B9	FACT complex subunit SPT16	SUPT16H	4.916804165	0.029706038	Chromatin factor that acts to reorganise nucleosomes
Q15428	Splicing factor 3A subunit 2	SF3A2	4.888994719	0.049833709	pre-mRNA splicing
P09234	U1 small nuclear ribonucleoprotein C	SNRPC	4.415730225	0.009614509	pre-mRNA splicing and assembly of Spliceasome
P09874	Poly [ADP-ribose] polymerase 1	PARP1	4.166542528	0.036637335	Poly-ADP-ribosyltransferase which plays a key role in DNA repair
Q7Z7K6	Centromere protein V	CENPV	3.962630426	0.012793511	Chromatin dynamics
P09382	Galectin-1	LGALS1	3.770809157	0.008733426	Plays a role in regulating apoptosis, cell proliferation and cell differentiation
P18124	60S ribosomal protein L7	RPL7	3.761747464	0.027984656	Component of large ribosomal subunit
P07910	Heterogeneous nuclear ribonucleoproteins C1/C2	HNRNPC	3.633218511	0.019705398	Modulates the translation of bound mRNA molecules
Q16778	Histone H2B type 2-E	HIST2H2BE	3.595164834	0.037766522	Core component of Nucleosome
Q13435	Splicing factor 3B subunit 2	SF3B2	3.519543657	0.009974206	pre-mRNA splicing
P12814	Alpha-actinin-1	ACTN1	3.13624407	0.024075282	F-actin cross-linking protein
Q9BZE4	Nucleolar GTP-binding protein 1	GTPBP4	3.040396777	0.021494321	Involved in the biogenesis of the 60S ribosomal subunit and role in cell cylcle arrest
Q99878	Histone H2A type 1-J	HIST1H2AJ	2.948961788	0.035223764	Core component of Nucleosome
P46821	Microtubule-associated protein 1B	MAP1B	2.90673827	0.046378376	microtubule dynamics and role in neuronal differentiation and neurite extension
Q15393	Splicing factor 3B subunit 3	SF3B3	2.776752911	0.005260514	pre-mRNA splicing
Q14498	RNA-binding protein 39	RBM39	2.711007929	0.038440466	pre-mRNA splicing
Q99879	Histone H2B type 1-M	HIST1H2BM	2.624807599	0.014717804	Core component of Nucleosome
O00159	Unconventional myosin-Ic	MYO1C	2.622863516	0.046613274	Unconventional myosins serve in intracellular movements
Q71UI9	Histone H2A.V	H2AFV	2.431466734	0.004555345	Variant Histone H2A replaces concventional H2A in a subset of nucleosomes
Q9NWH9	SAFB-like transcription modulator	SLTM	2.357582706	0.044995606	General inhibitor of transcription that eventually leads to apoptosis
P46777	60S ribosomal protein L5	RPL5	2.293661233	0.020821231	Component of the ribosome
P55769	NHP2-like protein 1	NHP2L1	2.200878055	0.005656749	pre-mRNA splicing
Q9UHB9	Signal recognition particle subunit SRP68	SRP68	2.181682188	0.018012305	Component of the SRP, targetting of secretory and membrane proteins to ER
P06748	Nucleophosmin	NPM1	2.064038125	0.016134266	Involved in diverse cellular processes
Q15717	ELAV-like protein 1	ELAVL1	2.030895413	0.027963344	binds to the 3'-UTR region of mRNAs and increases their stability
P40425	Pre-B-cell leukemia transcription factor 2	PBX2	2.016451131	0.009139004	Transcriptional activator

Supplementary Table 1 to show proteins enriched in tau immuno-precipitates after dGAE treatment. *Changes to the tau interactome in dSH cells were explored using co-immunoprecipitation combined with quantitative LC-MS as described in the materials and methods section. T test analysis was used to identify changes to the tau interactome. Proteins that were enriched two-fold or more after dGAE treatment with a P value of <0.05 were selected.*

#term ID	Term description	Observed gene count	Background gene count	Strength	false discovery rate
GO:0070013	Intracellular organelle lumen	37	5857	0.49	2.68E-13
GO:0032991	Protein-containing complex	33	5073	0.5	5.05E-11
GO:0031981	Nuclear lumen	32	4733	0.52	6.27E-11
GO:1990904	Ribonucleoprotein complex	15	677	1.03	7.10E-10
GO:0005634	Nucleus	35	7390	0.36	2.69E-08
GO:0005654	Nucleoplasm	27	3973	0.52	3.24E-08
GO:0005681	Spliceosomal complex	9	192	1.36	4.10E-08
GO:0005684	U2-type spliceosomal complex	7	94	1.56	2.15E-07
GO:0071005	U2-type precatalytic spliceosome	6	50	1.77	2.27E-07
GO:0043232	Intracellular non-membrane-bounded organelle	28	4880	0.45	3.60E-07
GO:0097525	Spliceosomal snRNP complex	6	56	1.72	3.60E-07
GO:0016604	Nuclear body	13	789	0.91	3.74E-07
GO:0043229	Intracellular organelle	40	12528	0.19	1.65E-06
GO:0005686	U2 snRNP	4	18	2.04	8.99E-06
GO:0005730	Nucleolus	12	924	0.8	1.71E-05
GO:0043231	Intracellular membrane-bounded organelle	37	10761	0.23	1.94E-05
GO:0043227	Membrane-bounded organelle	39	12427	0.19	2.35E-05
GO:0071013	Catalytic step 2 spliceosome	5	87	1.45	8.01E-05
GO:0016607	Nuclear speck	8	399	0.99	9.04E-05
GO:0032993	protein-DNA complex	6	195	1.18	0.00019
GO:0001651	Dense fibrillar component	2	2	2.69	0.0014
GO:0005689	U12-type spliceosomal complex	3	25	1.77	0.0014
GO:0070062	Extracellular exosome	14	2099	0.51	0.0023
GO:0000786	Nucleosome	4	106	1.27	0.0037
GO:0031428	Box C/D snoRNP complex	2	7	2.15	0.0068
GO:0005925	Focal adhesion	6	405	0.86	0.0079
GO:0005694	Chromosome	11	1712	0.5	0.0215
GO:0071004	U2-type prespliceosome	2	19	1.71	0.0353

Supplementary Table 2. A PPI network of the enriched proteins identified in a T test comparing dGAE treated to untreated control cells. *Proteins that were enriched two-fold or more after dGAE treatment with a P value of <0.05 were selected. The PPI network was generated for enriched proteins using the STRING database. Functional enrichments for the uploaded proteins are shown for Gene Ontology terms classified as cellular component.*

Protein names	Gene names	Fold Change_ Control high	Student's T-test p-value dGAE to Control	Abbreviated Function from Uniprot
Vacuolar protein sorting-associated protein 54	VPS54	1292.463922	0.01288001	Transport from Endosomes to Golgi network
Kallikrein-5	KLK5	22.07286009	0.037938769	May be involved in desquamation
Statherin	STATH	19.74561733	0.088392394	Inhibits precipitation of calcium phosphate salts
Proactivator polypeptide-like 1	PSAPL1	6.914502848	0.032962957	May activate the lysosomal degradation of sphingolipids
Y-box-binding protein 2	YBX2	5.215236328	0.014792386	Regulation of the stability and/or translation of germ cell mRNAs
Extracellular glycoprotein lacritin	LACRT	3.787071307	0.00563755	Modulates secretion by lacrimal acinar cells
Retroviral-like aspartic protease 1	ASPRV1	3.759237307	0.022400433	Protease responsible for filaggrin processing
Ig lambda chain V-III region LOI	IGLV3-9	3.700231877	0.027379345	Immunoglobulins
Lactotransferrin	LTF	3.416872202	0.041241683	Iron binding transport protein
Lysozyme C	LYZ	3.386256035	0.047736034	Lysozymes

Supplementary Table 3. Proteins enriched in tau immuno-precipitates from untreated control cells compared to dGAE treated cells. *Changes to the tau interactome in dSH cells were explored using co-immunoprecipitation combined with quantitative LC-MS as described in the materials and methods section. T test analysis was used to identify changes to the tau interactome. Proteins that wereenriched two-fold or more in control untreated cells compared to dGAE treated cells with a P value of <0.05 were selected.*
